# Knowledge and perceptions regarding Human Papillomavirus (HPV) and willingness to receive HPV vaccination among university students in a north-eastern city in Turkey

**DOI:** 10.1186/s12905-023-02455-4

**Published:** 2023-06-06

**Authors:** Ceren Varer Akpinar, Sebnem Alanya Tosun

**Affiliations:** 1 Department of Public Health, Giresun University Faculty of Medicine, Giresun, Turkey; 2 Department of Obstetrics & Gynaecology, Giresun University Faculty of Medicine, Giresun, Turkey

**Keywords:** Human papillomavirus, HPV vaccine, Vaccination, Knowledge, University students

## Abstract

**Background:**

For a HPV vaccination program to be successful, it is important that the target population has sufficient knowledge about HPV and HPV vaccines. The aim of this study was to evaluate HPV-related knowledge levels among students at a university in northern Turkey, to assess vaccination willingness, and to determine factors associated with HPV-related knowledge.

**Methods:**

This cross-sectional study was conducted on 824 (93.1%) students studying at 16 different faculties. The study population was identified through the proportional stratified sampling method. Data were collected through a questionnaire including socio-demographic characteristics and the HPV Knowledge Scale. Multiple linear regression analysis was performed to identify factors that may be associated with knowledge scores.

**Results:**

43.6% of the students had never heard of HPV, 50.6% of them had never heard of HPV screening tests or HPV vaccination. Only 2.7% of the students were vaccinated against HPV and 15.7% were willing to receive HPV vaccination. Awareness of HPV and willingness to receive vaccination were found to be higher among women, while previous experience with sexual intercourse was higher in men (*p* < 0.05). The mean HPV knowledge score was quite low (6.74 ± 7.13 out of 29 points). The studying in a field related to health sciences, being a senior student, intending to be vaccinated, being a woman, having had sex were found to be associated with high knowledge levels (*p* < 0.05).

**Conclusions:**

Educational programmes should be developed to increase university students' knowledge about HPV and the HPV vaccine.

**Supplementary Information:**

The online version contains supplementary material available at 10.1186/s12905-023-02455-4.

## Background

Human Papillomavirus (HPV) infection is one of the most frequently encountered sexually transmitted infections [1]. It is estimated that about 90% of sexually active men and 80% of sexually active women will be infected with various HPV types during their lives [2]. Fortunately, 90% of HPV infections disappear within 1–2 years without any symptoms, while 10% cause diseases. Among the disease-causing subtypes, low-risk genotypes (especially HPV 6 and 11) cause genital warts; whereas high-risk genotypes (especially HPV 16 and 18) are responsible for about 70% of all cervical cancer cases worldwide and are also being associated with head and neck and anogenital cancers [3]. Current estimates show that cervical cancer is the 4th most frequent cancer among women in the world (incidence: 15.6 per 100,000) [4], while it is the 9th most common cancer among women in Turkey (incidence: 4.2 per 100,000) [5].

In addition, in Islamic countries with a traditional culture such as Turkey, it may result in significant psychological consequences due to the sexually transmitted nature of the virus [6, 7]. In studies HPV infections result was associated with stigma, distress, unrealistic fear of cancer, and concerns over deteriorating of emotional relationships [6, 8, 9].

With the development of vaccines against critical HPV types (types 16 and 18), vaccination has become the primary preventative measure [10]. With the development of HPV vaccines, discussions about HPV vaccine and HPV-related diseases started in Turkey as well as in other countries of the world. In 2009, the World Health Organization (WHO) recommended the inclusion of HPV vaccination in national immunization programs [11]. At the end of 2020, WHO announced a global strategy to accelerate the elimination of cervical cancer as a public health problem. Three main targets have been set to be achieved by 2030: vaccination (HPV vaccination of 90% of girls by the age of 15), screening (70% of women screened using a high-performance test by the age of 35, and again by the age of 45), and treatment (90% of women with pre-cancerous lesions treated and 90% of women with invasive cancer managed) [12, 13]. As of 2020, more than 100 countries have included HPV vaccinations in their national immunization programs [14, 15]. The bivalent, quadrivalent and 9-valent HPV vaccines are commercially available in Turkey but have not been included in the national immunization program.

Receiving HPV vaccination before sexual activity and before the first exposure to HPV is crucial [16, 17]. Although the age of first sexual intercourse varies according to countries and cultures, university age is generally accepted as the beginning of sexual activity in Turkey. There is growing evidence that sexually transmitted infections are on the rise among university students, and thus, it is clear that these students represent a high-risk group for HPV infection [18].

In order to carry out a successful HPV vaccination program, the target population should have adequate knowledge about HPV and HPV vaccines. Therefore, determining the level of HPV-related knowledge in the university student population can guide interventions and policies aimed at eliminating HPV-related cervical cancer. In this study, we aimed to evaluate knowledge and perceptions about HPV and HPV vaccines among students studying at Giresun University, to assess their willingness to be vaccinated, and to investigate factors associated with HPV-related knowledge.

## Materials and methods

### Design, participants and sampling

This cross-sectional study was conducted in Giresun University and included undergraduate students between February 2022 and April 2022. No special policies or news about HPV or HPV vaccination were released by the Ministry of Health or other organizations during the study period. The study population was to be selected from an overall pool of 9443 students enrolled in 16 faculties of the university. The pilot study was conducted in 20 participants to evaluate the comprehensiveness and language of the questionnaire. A target sample size of 885 students was determined using 50% prevalence with 5% error margin, 95% confidence interval, design effect 2 and 20% non-response rate. In sample selection, students were stratified according to their faculty first and then according to their grade level (years) in each faculty. The proportional stratified sample selection method was employed to select subjects in proportion for each faculty in the student pool. After the faculties were stratified according to grade level, simple random selection was made from each class list. The inclusion criteria for the study are as follows: to be a current student at the relevant university, to be native speakers to complete the questionnaire form in full. Participants filled out the questionnaire in-person with the self-report method. A total of 885 questionnaires were handed out, and the response rate was 93.1% (*n* = 824).

### Instrument

The first part of questionnaire was designed by researchers, including 15 questions related to socio-demographic characteristics, medical history, sexual life and knowledge regarding HPV infection and vaccination. The second part of questionnaire was the HPV Knowledge Scale (HPV-KS), which is a 35-item scale developed by Waller et al. to measure knowledge about HPV, HPV vaccination and screening tests. It includes three subscales concerning general information regarding HPV infection/treatment, HPV screening, and HPV vaccination, while there is also an independent 6-item subscale [19]. The Turkish validity and reliability study of the scale was conducted by Demir et al. [20]. Cronbach's alpha value of the scale is 0.96. The first subscale includes questions about HPV risk factors, transmission route, prevention methods, general information about treatment of HPV infection; the second subscale assesses knowledge regarding pap smear and HPV DNA testing for cervical cancer screening; the third subscale evaluates knowledge about HPV vaccines and related diseases. Finally, the 6-item independent subscale examines knowledge status about the current vaccination schedule (in countries including HPV in their immunization programs). Since HPV vaccination is not included in the immunization program in our country, this last subscale was not applied. Each item in the questionnaire has three possible options (Yes/No/Don’t know). All items have a correct answer in the form of “Yes” or “No”; thus, the “Don’t know” option is always scored as an incorrect answer. Correct answers are scored as 1 point; incorrect answers are scored as 0 points. The scale has no breakpoints. Total scores range from 0 to 29, with higher scores indicating greater knowledge. Cronbach’s alpha value of the scale was 0.94 for the current study.

### Ethical considerations

All steps of the study conformed to the Declaration of Helsinki and the study protocol was approved by the Institutional Review Board of Ordu University (January 19, 2022, no: 0685189). Additionally, permission to conduct the study on university students was obtained from the university administration.

Before completing the questionnaires, the participants were well informed about the purpose and contents of the study. Participants were asked to sign informed voluntary consent forms. To prevent response bias, participants were guaranteed anonymity and their personal information was kept strictly confidential. After data collection was completed, an informative online booklet about HPV and HPV vaccines containing the correct answers to the questions used to measure their knowledge levels was sent to the students via their university e-mail addresses.

### Statistical analysis

Study data were evaluated using the SPSS 24.0 statistics program. Since much of the literature relies on data from studies conducted on females, many analyses were stratified according to sex. For descriptive analyses, numeric variables were described with mean and standard deviation, categorical variables were described as absolute and relative (%) frequencies. Normal distribution of data was evaluated with the Kolmogorov–Smirnov test, histograms, and skewness and kurtosis coefficients. According to these results, the independent samples t-test and Kruskal Wallis tests were used to evaluate relationships between HPV knowledge levels and independent variables. Multiple linear regression analysis was performed to evaluate factors independently associated with HPV knowledge scores. The aim was to eliminate the effect of confounding factors with multiple regression analysis. Variables that were found to be related with HPV knowledge scores in univariate analyses were included in the regression model. Of note, 33 data identified by their Mahalanobis values before regression were not included in the analysis. Significance level was accepted as *p* < 0.05, and 95% confidence intervals were calculated.

## Results

A total of 824 university students participated in the study. Table 1 shows the socio-demographics characteristics of participating students. The mean age of students was 21.4 ± 3.4 years. They were from 16 different faculties/vocational schools. The study group included students from 68 of the 81 provinces in Turkey.

**Table 1:** Socio-demographic characteristics of participating students (*n *= 824)

Variables	n	%
Sex		
Female	512	62.1
Male	312	37.9
Region		
West	164	19.9
South	94	11.4
Middle	85	10.3
East	127	15.4
North	354	43.0
Place of family’s residence		
Rural	682	82.8
Urban	142	17.2
Faculty		
Health sciences	132	16.0
Others	692	84.0
Class level		
1	182	22.1
2	211	25.6
3	197	23.9
≥ 4	234	28.4
Perception of income		
Good	160	19.4
Average	551	66.9
Bad	113	13.7

The medical / sexual histories and HPV-related characteristics of participants are described in Table 2. Overall, 24.4% of students stated that they had experienced sexual intercourse. Mean age at first sexual intercourse was 19 ± 2.7 years (females: 22.2 ± 4.7, males: 18.8 ± 2.0). Among students, 43.6% had never heard of HPV, 50.6% had never heard of HPV screening tests and HPV vaccination. Among students, only 2.7% had been vaccinated against HPV and only 15.7% were willing to receive HPV vaccination. A family history of genital cancer, visiting a physician in the last year, being aware of HPV/HPV screening tests and/or HPV vaccination, and willingness to get vaccinated were found to be higher in females, whereas males were more likely to have had sexual intercourse before the study (*p* < 0.05) (Table 2).

**Table 2:** Medical and sexual history and HPV related characteristics of participating students (Overall and stratified for sex) (*n *= 824)

Variables	Total		Female		Male		*P*
	**n**	**%**	**n**	**%**	**n**	**%**	
History of genital cancer in the family							
Yes	26	3.2	22	**4.3**	4	1.3	**0.011**
No	798	96.8	490	**95.7**	308	98.7	
Ever had sex before							
Yes	201	24.4	69	13.5	132	**42.3**	**<0.001**
No	623	75.6	443	86.5	180	**57.7**	
Age at first sexual intercourse^a^							
16–18	72	39.3	8	11.6	64	**56.1**	**<0.001**
≥ 19	111	60.7	61	88.4	50	**43.9**	
Physician visit within last year							
Gynecologist/urologist	49	5.9	44	**8.6**	5	1.6	**<0.001**
Other physician	534	64.8	336	**65.6**	198	63.5	
No	241	29.2	132	**25.8**	109	34.9	
Having heard about HPV							
Yes, has sufficient knowledge	114	13.8	86	**16.8**	28	9.0	**<0.001**
Yes, does not have sufficient knowledge	351	42.6	251	**49.0**	100	32.1	
No	359	43.6	175	**34.2**	184	59.0	
Having heard about HPV screening tests							
Yes, has sufficient knowledge	95	11.5	68	**13.3**	27	8.7	**<0.001**
Yes, does not have sufficient knowledge	312	37.9	223	**43.6**	89	28.5	
No	417	50.6	221	**43.2**	196	62.8	
Having heard about HPV vaccination							
Yes, has sufficient knowledge	125	15.2	92	**18.0**	33	10.6	**<0.001**
Yes, does not have sufficient knowledge	282	34.2	214	**41.8**	68	21.8	
No	417	50.6	206	**40.2**	211	67.6	
Having received HPV vaccination							
Yes	22	2.7	18	3.5	4	1.3	0.059
No	802	97.3	494	96.5	308	98.7	
Willing to receive HPV vaccination							
Yes	129	15.7	104	**20.3**	25	8.0	**<0.001**
No idea	363	44.1	217	**42.4**	146	46.8	
No	332	40.3	191	**37.3**	141	45.2	

^a^Percentage calculated among students who had had sex before and were willing to state when

**Fig. 1:**
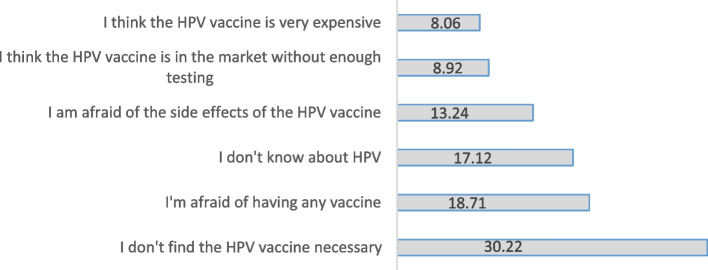
Reasons associated with refusal of vaccination against HPV (%) (*n* = 695)

Figure 1 shows the reasons for refusal of HPV vaccination by the students. The most common reasons were the following: Believing HPV vaccination was unnecessary (30.2%), being afraid of any vaccine (18.7%), not knowing about HPV (17.1%).

The average mean score of the students’ HPV-KS was 6.74 ± 7.13 (highest score possible: 29 points). General HPV knowledge mean score was 4.68 ± 4.42, HPV screening test knowledge mean score was 0.80 ± 1.38, and HPV vaccination knowledge mean score was 1.25 ± 1.92 points. HPV knowledge scale total score and subscale mean scores were significantly higher in women (*p* < 0.05) (Table 3).

**Table 3:** Human papillomavirus knowledge scale and subscale mean scores of participating students (Overall and stratified for sex) (*n* = 824)

	**Total**	**Female**	**Male**	***P*** *
	**Mean ± SD**	**Mean ± SD**	**Mean ± SD**	
HPV-KS (max score: 29)	6.74 ± 7.13	7.18 ± 7.47	6.01 ± 6.50	**0.012**
The general HPV knowledge (max score: 16)	4.68 ± 4.42	4.90 ± 4.54	4.31 ± 4.20	**0.041**
The HPV screening test knowledge (max score: 6)	0.80 ± 1.38	0.87 ± 1.44	0.68 ± 1.27	**0.042**
The general HPV vaccination knowledge (max score: 7)	1.25 ± 1.92	1.40 ± 2.05	1.01 ± 1.65	**0.003**

^*^Student t-test

With the HPV knowledge scale and subscale scores of the students; a relationship was found between sex, faculty, grade level, income, sexual intercourse status, doctor visit, HPV vaccine willingness (*p* < 0.05) (Supplementary Table S1).

To clarify parameters independently associated with HPV knowledge levels, we created a multiple linear regression model by including variables that were found to be statistically significant in univariate analyses. The model generated was significant (F = 19.70, *p* < 0.001); together with the relevant variables, it explained 42% of the change in HPV knowledge (adjusted R^2^ = 0.418). According to the regression analysis, being a female, studying health sciences, being a 4^th^ year student, to have had sex previously, having visited a gynecologist / urologist within the last year, and being willing to receive HPV vaccination were identified as factors that were independently associated with higher levels of HPV knowledge (*p* < 0.05) (Table 4).

**Table 4:** Multiple linear regression analysis of factors associated with HPV knowledge

Variable	B	Std. Error	β	t	*p*	95% CI	
Sex (Female)	2.52	0.43	0.17	5.75	< 0.001	1.66	3.38
Faculty (Health Sciences)	7.23	0.57	0.36	12.47	< 0.001	6.00	8.25
Class, years (4th year)	3.27	0.44	0.20	7.29	< 0.001	2.39	4.15
Perception of income (good/very good)	0.46	0.51	0.02	0.90	0.36	-0.54	1.46
Ever had sex (Yes)	2.06	0.49	0.12	4.15	< 0.001	1.08	3.03
Physician visit within last year (Gynecologist/urologist)	1.82	0.85	0.06	2.13	< 0.03	1.14	3.50
Willing to receive HPV vaccination (Yes)	4.18	0.60	0.21	6.95	< 0.001	3.00	5.36

*R* ^2^:0.418 f:19.70 *p* < 0.00

## Discussion

This study evaluated the HPV and HPV vaccination-related knowledge levels of Turkish university students, who are the target population for HPV vaccination due to the estimated age of first sexual experience.

Approximately one-fourth of the students in the study group stated that they had sexual intercourse before, but men had almost three times greater frequency than that of women. In our study, the average age of first sexual intercourse was 19, but this value was higher among women. The age at first sexual intercourse differs with respect to countries and cultural contexts. The average age of sexual intercourse is around 19 in some western European countries, 17 in China and 16 in the United States. However, in these studies, no sex-based difference was observed in terms of the age of first sexual experience [21–23]. Despite the changing attitudes under the influence of western culture in Turkey, sexual intercourse at an early age and before marriage is not approved in traditional culture [24]. This view is generally dominant for women in the context of sex inequality. This may explain the sex-based difference in the frequency of having experienced sexual intercourse in our study.

In our study group, 44% of participants had never heard of HPV, 51% had never heard of HPV screening tests or HPV vaccines. These rates were lower in women than in men. In a study conducted with university students in China, 49% of them stated that they had never heard of HPV and 51% of them stated that they had never heard about the HPV vaccine, and this rate was found to be significantly lower in women than in men similar to our study [22]. Another study conducted in Thailand showed that 73.8% of male students and 69.6% of female students did not know who should be screened for cervical cancer [25]. In studies conducted in European countries and the USA, the rate of hearing about HPV and HPV vaccine was reported as 82–90%, which is considerably higher than our study results. However, similar sex-based differences were also shown in these studies [26–29]. In most western countries, HPV vaccination is part of the national vaccination program, especially for females, and training on HPV is provided during school years. HPV vaccine is not yet included in the national vaccination program in Turkey and there is no HPV-related program in the education curricula. Studies confirm that education about HPV will increase the level of HPV knowledge [30, 31]. This difference between countries may be due to the training activities carried out within the scope of the national immunization program.

Vaccination rates against HPV are very low in the study group. Only 2.7% were vaccinated against HPV. In many European countries, almost 80% of the target population has been vaccinated [32]. In a study conducted with female university students in Poland, a country which does not have a HPV immunization program, vaccination rates against HPV were below 10% [33]. Lower vaccination rates may be related to the lack of government-supported subsidy programs for HPV vaccination or inclusion in national immunization programs. It is expected that higher vaccination rates will be observed in countries that have included the HPV vaccine in their vaccination program.

In the present study, while 16% of participants were willing to be vaccinated, 40% were undecided about HPV vaccination, and 44% did not want to be vaccinated. While the desire to be vaccinated was higher in women, indecision was similar for both sexes. There are a wide variety of behavioral approaches from country to country to improve HPV vaccination intent. In a study conducted in Hong Kong, 13.3% of individuals were vaccinated against HPV and 69.6% of students stated that they planned to be vaccinated [34]. A similar study conducted in Korea similarly reported a 62.8% intention to vaccinate among participants [35]. In general, the willingness to be vaccinated is higher in western countries [32]. The high frequency of students unwilling to vaccinate in our study group may be related to low level of knowledge or being used to receiving free-of-charge vaccination by the government. Therefore, in addition to the lack of information, relatively high vaccination costs when met with sufficient information may lead to low rates of vaccination against HPV. Other studies have also reported that the costs associated with HPV vaccines may be an obstacle to HPV vaccination [36, 37]. In developing countries such as Turkey, where the HPV vaccine is not included in the national program, HPV vaccine becomes a privilege that can be accessed by those with higher socioeconomic status.

Although almost half of the students included in this study were aware about HPV, HPV screening tests and HPV vaccination; however, their knowledge scores were unfortunately quite low. The level of knowledge about HPV was lower compared to studies conducted in developed countries [26, 28, 29, 34]. The low level of knowledge is probably related to the limited importance given to HPV in education programs in Turkey. Awareness trainings are insufficient in terms of content and frequency. This might result from the traditional Turkish cultural context, which fosters a conservative attitude toward sex issues. In an intervention study conducted with high school students in Sweden, it was shown that knowledge significantly increased among male and female students after training on HPV and HPV prevention [38]. In this context, before the HPV vaccine is added to the national immunization program, it may be important to include education programs on sexually transmitted diseases and HPV into the education curriculum.

In our study, knowledge scores on HPV, HPV screening and HPV vaccine were found to be higher in women. In the literature, similar studies conducted with the participation of students from countries with different levels of development have shown that women generally have better knowledge and attitudes towards HPV than men [22, 26, 30]. The low level of knowledge about HPV and HPV vaccination in men with sexual experience at an earlier age increases the risk of HPV transmission [39]. Health promotion efforts to prevent HPV infection have focused primarily on cervical cancer which is unrelated to male health. Therefore, male students may be less aware of the potential impact of HPV infection on them and the benefits of vaccination.

This study also showed that students studying in non-health-related programs have less knowledge about HPV than students studying in health-related faculties/vocational schools. Studies conducted on students in the field of health sciences have shown that they have more awareness about HPV and HPV vaccines [40, 41]. A study conducted in Portugal revealed that students studying in health science departments were more likely to have heard about HPV than students from other departments [42]. It is to be expected that students in health-related fields will learn more about HPV infection and cervical cancer. Significantly higher scores among students in health-related programs may mean that better education is effective in improving students' knowledge and attitudes about HPV and HPV vaccination. However, the level of knowledge about general health, such as HPV, should be at a certain level in all young people, regardless of their field of education. In addition, in our study, the level of knowledge about HPV, HPV screening and HPV vaccination was found to be higher in senior students, regardless of the faculty they studied. In the international literature, it was stated that the level of knowledge about HPV increases with increasing age among students [43]. In our study, knowledge levels about HPV, HPV screening and HPV vaccines were found to be higher in those who had sexual intercourse before. In another study, the level of knowledge of those with sexual experience was found to be higher [44]. HPV vaccines are established to be most effective when administered before the onset of sexual activity. Sexually active people may benefit less because they may have already been exposed to one or more of the HPV types targeted by the vaccine. For this reason, it is important to provide information about HPV and HPV vaccination at an early age, particularly before first sexual experience.

Willingness to receive the HPV vaccination was significantly associated with knowledge scores for HPV for both male and female participants. This study shows that the level of knowledge is higher among those who are willing to be vaccinated. This is consistent with previous studies that found an association between greater knowledge of HPV and HPV vaccines and intention to vaccinate [22, 34, 45–47]. Knowledge is an important indicator of willingness to vaccinate and vaccination rates. Therefore, education to increase knowledge about HPV and HPV vaccines will increase both willingness to be vaccinated and vaccination rates.

Most studies on HPV and HPV vaccination in Turkey generally focus only on women, and few studies have analyzed HPV-related knowledge in both males and females. This study has the advantages of obtaining a good sample size with appropriate sample selection, lack of refusal in participation, and that the selected group represented students who originated from the great majority of the country’s provinces. In addition, a scale with proven validity and reliability was used to evaluate the level of knowledge. However, there are some limitations of our study. First, the population of this study is not representative of the general youth population in Turkey, as it consists of university students. Second, we should take caution when interpreting the findings of our study, because a drawback of the cross-sectional study design is the lack of causality assessment.

## Conclusion

Even among university students, who are a more educated group in Turkey, knowledge of HPV and HPV vaccination is at a very low level. In addition, HPV vaccination rates and vaccination intent appear to be very low. Despite having sexual experience at an earlier age, the knowledge level of men about HPV and HPV vaccine is lower. Students who have higher knowledge about HPV and HPV vaccine have a higher willingness to vaccinate.

The results of this study show that there is a need for education programs on HPV infection and vaccination. Studies should be conducted on the effectiveness of HPV-related educational interventions on willingness to be vaccinated. Before inclusion of HPV vaccination in the national immunization program, it is necessary to carry out health promotion studies and policies in order to improve the knowledge and attitudes regarding HPV and HPV vaccination, particularly for young men. The results of these studies should be taken into account when developing interventions and policies to eradicate HPV-related cervical cancer in countries where the HPV vaccine is not included in the national immunization program.

## Supplementary Information

**Additional file 1:** **Supplementary Table S1.**

## Data Availability

The datasets used and/or analysed during the current study are available from the corresponding author on reasonable request.

## Abbreviations

HPV: Human papillomavirus
WHO: World health organization
FDA: Food and drug administration
