# Supplementary material for: Knowledge and perceptions regarding Human Papillomavirus (HPV) and willingness to receive HPV vaccination among university students in a north-eastern city in Turkey
Source: BMC Womens Health. 2023 Jun 6;23:299. doi: 10.1186/s12905-023-02455-4 (PMC10243033; doi:10.1186/s12905-023-02455-4)
Supplement: Supplementary file 1 — Additional file 1: Supplementary Table S1. [file 12905_2023_2455_MOESM1_ESM.docx]

**Supplementary Table S1**

|  | **HPV knowledge**  **(total)** | | **General HPV knowledge** | | **HPV screening test**  **knowledge** | | **HPV vaccination**  **knowledge** | |
| --- | --- | --- | --- | --- | --- | --- | --- | --- |
|  | **Mean±SD** | **Test and Significance** | **Mean±SD** | **Test and Significance** | **Mean±SD** | **Test and Significance** | **Mean±SD** | **Test and Significance** |
| **Sex**  Female  Male | 7.18±7.47  6.01±6.50 | T= 2.37  **P=0.010** | 4.90±4.54  4.31±4.20 | T=1.98  **P=0.041** | 0.87±1.44  0.68±1.27 | T=1.99  **P= 0.042** | 1.40±2.05  1.01±1.65 | T=2.98  **P=0.003** |
| **Region**  West  South  Central  East  North | 6.83±6.33  6.53±6.35  6.43±6.08  6.70±6.18  6.64±6.59 | KW=2.54  P=0.323 | 5.09±4.59  5.76±4.08  5.32±4.60  5.00±.439  5.13±4.22 | KW=3.87  P=0.230 | 0.75±1.29  0.78±1.81  0.78±1.27  0.78±1.41  0.74±1.22 | KW=5.67  P=0.081 | 1.39±1.82  1.21±1.42  1.31±2.02  1.52±1.91  1.40±1.67 | KW=3.10  P=0.203 |
| **Place of family’s residence**  Rural  Urban | 6.59±7.05  6.77±7.16 | T=-0.28  P=0.772 | 4.79±4.63  4.66±4.38 | T=-0.32  P=0.743 | 0.78±1.27  0.80±1.40 | T=-0.19  P=0.842 | 1.01±1.68  1.30±1.97 | T=-1.66  P=0.071 |
| **Faculty**  Health Sciences  Others | 14.31±8.33  5.30±5.87 | T=15.00  **P<0.000** | 8.87±4.59  3.88±3.91 | T=11.70  **P<0.000** | 2.21±1.94  0.53±1.05 | T=14.39  **P<0.000** | 3.21±2.40  0.88±1.56 | T=14.24  **P<0.000** |
| **Class (year)**  1  2  3  ≥4 | 2.36±3.27  5.55±5.48  6.07±6.68  9.97±8.35 | KW= 111.89  **P<0.000** | 2.05±2.69  3.29±3.13  4.32±4.19  6.47±4.92 | KW=103.24  **P<0.000** | 0.09±0.36  0.51±1.06  0.71±1.27  1.41±1.81 | KW=95.74  **P<0.000** | 0.21±0.73  0.80±0.87  1.03±1.79  2.08±2.27 | KW=116.11  **P<0.000** |
| **Perception of income**  Good  Average  Bad | 8.59±7.59  6.64±7.22  4.62±5.19 | KW=18.79  **P<0.000** | 6.06±4.64  4.48±4.41  3.69±3.67 | KW=21.93  **P<0.000** | 0.98±1.46  0.83±1.43  0.38±0.84 | KW=13.63  **P=0.012** | 1.55±2.17  1.31±1.93  0.54±1.25 | KW=20.13  **P<0.000** |
| **History of genital cancer in the family**  Yes  No | 5.65±7.71  6.78±7.12 | T=-0.79  P=0.421 | 3.92±4.50  4.70±4.42 | T=-0.89  P=0.379 | 0.65±1.09  0.80±1.39 | T=-0.55  P=0.573 | 1.07±2.27  1.26±1.91 | T=-0.48  P=0.628 |
| **Ever had sex**  Yes  No | 8.25±7.42  6.25±6.98 | T=3.48  **P= 0.001** | 5.80±4.56  4.32±4.32 | T= 4.15  **P<0.000** | 1.01±1.52  0.7±1.32 | T=2.28  **P=0.023** | 1.44±1.92  1.19±1.92 | T=3.60  **P=0.012** |
| **Age at first sexual intercourse**  16-18  **≥19** | 7.56±6.83  8.92±7.61 | T=-1.22  P=0.212 | 5.47±4.30  6.18±4.60 | T=-1.04  P=0.290 | 1.00±1.61  1.03±1.48 | T=-0.15  P=0.800 | 1.09±1.69  1.71±2.01 | T=-1.13  P=0.051 |
| **Physician visit within last year**  Gynaecologist/urologist  To other doctor  No | 10.79±8.83  6.53±6.79  6.38±7.28 | KW=12.74  **P=0.002** | 7.22±4.80  4.64±4.27  4.26±4.52 | KW=15.70  **P=0.010** | 1.36±1.84  0.75±1.32  0.79±1.38 | KW=7.68  **P=0.010** | 2.20±2.66  1.13±1.83  1.33±1.89 | KW=7.99  **P=0.010** |
| **Willing to receive HPV vaccination**  Yes  No idea  No | 13.72±7.19  4.62±6.30  6.20±6.27 | KW=142.70  **P<0.000** | 8.75±3.84  3.21±4.06  4.57±4.03 | KW=138.34  **P<0.000** | 1.90±1.73  0.52±1.15  0.66±1.24 | KW=117.81  **P<0.000** | 3.06±2.24  0.87±1.58  0.96±1.72 | KW=124.44  **P<0.000** |
